# Temperature, Food, and Dress

**Published:** 1893-08-26

**Authors:** 


### The Hospital

An Institutional Journal of

Science, Medicine, Nursing, and Philanthropy.

*For Hospitals, Asylums, Infirmaries, Dispensaries, Medical Practitioners, Students, Nurses, and the Charitable Public.*

Vol. XIV.—No. 361.] [August 26, 1893.

### Temperature, Food, and Dress.

For years the average Englishman has complained bitterly that the climate of the British Isles was going to the dogs. This contention is based upon the facts that year after year the sun has made its influence less and less felt, and the number of hours of sunshine during each season have been so few that most outdoor occupations were rendered unattractive, if not uncomfortable. An increase in the rainfall, a diminution in the hours of sunshine, and a general and continuous fall in the temperature have each year tended to emphasise the discomforts of a temperate climate like that of England. Yet the history of the world proves that the English race, from the circumstances of their climate, are the hardiest in the world, and certainly the most capable of adapting themselves to the climacteric conditions of other countries with the minimum of discomfort and risk to themselves. The present year has been phenomenal because, almost for the first time in the memory of the present generation, we have enjoyed abundance of sunshine and all the delights of continuous fine weather. During the present month the temperature has been high, with a tendency to increase, and on the 18th inst. it reached a maximum of 98 deg. in the shade, the hottest day by far in 36 previous years having been July 15th, 1881, when the shade temperature was 97·1 at Greenwich. On the 18th inst. a very high rate of temperature was maintained throughout the 24 hours, it having been 84·3 in the shade at nine a.m., or 3·5 deg. higher than in any previous reading in 36 years. At eleven a.m. it had risen to 90·1, at noon it was 92·9, and at half-past one p.m. 93·6 degrees in the shade at Camden Town. The remarkable character of this heat is clearly proved by the fact that in the 36 years, 1858 to 1893, the shade temperature at nine a.m. has reached or exceeded 80 deg. on only nine days, and the shade maximum has reached or exceeded 90 deg. on 29 days only.

In these circumstances it is not surprising that some alarm should have been excited, and that the newspapers should have been full of complaints and warnings. Yet those who had common sense and a power of adapting themselves to the altered conditions which the higher temperature indicated, have been on the whole more comfortable and happy in the sunshine of 1893, and during the heat of the past few weeks, than they have probably ever been before in their lives. In a dry climate like England, a high temperature with abundance of sunshine is not only endurable, but enjoyable too. Of course if people persist in wearing tall hats and excessive clothing, if they partake of abundance of food and an unlimited amount of liquid, they necessarily become more and more uncomfortable as the heat progresses. These discomforts are heightened by incautious exposure to the rays of the sun during the hottest portion of the day, and by an excessive amount of exertion, which the inhabitants of the Tropics would regard anyone as mad to attempt. On the whole we are inclined to believe that the extra sunshine has lessened, if it has not destroyed, the risks of cholera, and that it will tend to improve the health of every man and woman who have been wise enough to adapt themselves to the altered conditions of the temperature.

The precautions are few and simple which will enable the majority of people to enjoy continuous fine hot or cold weather in England to-day. First and foremost we must alter our system of diet. It is too little understood that the temperature of a healthy person varies very slightly so long as sound health is maintained. This temperature, 98·4 F., does not vary even with the season; it is the same in summer and winter. In summer it is kept down by the perspiration which, evaporating from the surface of the body, extracts heat from the body for the purpose of carrying on such evaporation. In winter it is raised by extra clothing and fires, and in default of this by the heat given off in the various chemical changes carried on within the body, always more active during the winter. This explains why more food can be taken in winter than in summer, the extra supply being required for the combustion within the body, to keep up the temperature. Conversely, less food should be taken in summer, and that food should consist mainly of simple diet, bread and butter, milk, sound ripe fruit, vegetables, meat sparingly, after the heat of the day is over, and non-stimulating drinks, taken slowly. There is probably little harm in the light beers, or claret or still Moselle and water at dinner, but the best drink in our opinion is lemon water, made by infusing the thin outer rind of the lemon, containing the essential oils, *i.e.*, the peel of two lemons to a pint of water, which should then be put in the ice safe and drunk cold. It is further desirable to avoid taking liquids except at meals, a ripe pear or other fruit being taken when the thirst is excessive, and to defer drinking the lemon water until the end of the meal.

Another essential precaution in hot weather is to wear the lightest of gauze merino underwear for the body, linen or cotton for the legs, and outside stuffs light in weight and colour, and loose woven. We take it that flannel is, on the whole, the most comfortable material to wear in hot as in cold weather, and we would strongly advise everybody during the former season to wear a straw or felt hat, and to avoid the top hat as one would shun poison. Above all it is important, as an American writer says, to take life easily, to make haste slowly, to keep the body tranquil and the mind serene. It is essential to rise early, and to do as much as possible of the day's work before the sun is high. If we English people were accustomed to a high temperature we should not continue to think it unmanly to enjoy an hour's light sleep in a darkened room during the hottest portion of the day, as we should find that this habit in tropical weather would keep the internal temperature down better than anything else. Having such weather, we should imitate the life of the Tropics. Excitement, hurry, undue exertion, the direct rays of the sun, strong drinks, much meat, and substantial meals at midday must be avoided. With such precautions a cheerful spirit will be cultivated, good temper will prevail, and everyone will soon come to see what a blessing hot weather, with its glorious sunshine and balmy breezes, undoubtedly is to the healthy, if only they have the wit to learn to enjoy it to the full. Englishmen are apt to take life too seriously, but seriousness does not agree well with a high temperature. Those of us whose work keeps us in cities may cheerfully and contentedly labour on, providing we modify the conditions to suit the altered circumstances. All that has been said applies with added force to the life of town residents, and they will be well advised to work quietly, to do no more than is absolutely necessary, and to get out into the country where there is air and shade as much as possible.

The tropical weather is most trying, of course, to the poor in great cities. The Earl of Meath and others have done much by the creation of open spaces to lessen this evil, and anyone who passes through the parks will find abundant evidence that the poor do very largely avail themselves of the facilities for an air bath which the public gardens and open spaces offer. Taken altogether, every class ought to welcome the continued fine weather despite the heat, and we make little doubt that, as a matter of fact, most of them do so. Medically speaking, if the simple precautions we have indicated are followed, the risks arising from the weather will prove to be slight indeed. The adult population will be free from discomfort in exact proportion to the amount of common sense they exhibit in regard to clothing, food, and hours of work. It is surprising that the river has been largely deserted during the past few weeks, as a boat offers the most pleasant means of taking an air bath imaginable. In the United States every office has its electrical fan, and we have no doubt, should the hot weather continue to favour these islands with its presence, that every Englishman will very soon adapt himself to the altered circumstances, and so as a nation we shall come to talk less about weather and liver, and more about enjoyment and rest.

